# Effect of chitooligosaccharide on the inhibition of SARS-CoV-2 main protease

**DOI:** 10.1186/s40824-023-00351-4

**Published:** 2023-02-17

**Authors:** Qian Wang, Yuanyuan Song, Mungu Kim, Sei Kwang Hahn, Ge Jiang

**Affiliations:** 1 Bioengineering College, Dalian University, 10 Xuefu Street, Jinzhou District, Dalian, 116600 Liaoning China; 2 Department of Materials Science and Engineering, Pohang University of Science and Technology (POSTECH), 77 Cheongam-Ro, Nam-Gu, Pohang, 790-784 Gyeongbuk Korea

**Keywords:** SARS-CoV-2, Mpro, Chitooligosaccharide, Spectroscopy, Molecular docking

## Abstract

**Background:**

The main protease (Mpro) is a crucial target for severe acute respiratory syndrome coronavirus (SARS-CoV-2). Chitooligosaccharide (CS) has broad-spectrum antiviral activity and can effectively inhibit the activity of SARS-CoV. Here, based on the high homology between SARS-CoV-2 and SARS-CoV, this study explores the effect and mechanism of CS with various molecular weights on the activity of SARS-CoV-2 Mpro.

**Methods:**

We used fluorescence resonance energy transfer (FRET), UV–Vis, synchronous fluorescence spectroscopy, circular dichroism (CD) spectroscopy and computational simulation to investigate the molecular interaction and the interaction mechanism between CS and SARS-CoV-2 Mpro.

**Results:**

Four kinds of CS with different molecular weights significantly inhibited the activity of Mpro by combining the hydrogen bonding and the salt bridge interaction to form a stable complex. Glu166 appeared to be the key amino acid. Among them, chitosan showed the highest inhibition effect on Mpro enzyme activity and the greatest impact on the spatial structure of protein. Chitosan would be one of the most potential anti-viral compounds.

**Conclusion:**

This study provides the theoretical basis to develop targeted Mpro inhibitors for the screening and application of anti-novel coronavirus drugs.

## Background

Pneumonia caused by the SARS-CoV-2 infection, with a long incubation period and high infectivity, poses a great threat to public health worldwide [1, 2]. Currently, antiviral drugs such as ribavirin and ritonavir, which are used to treat patients with atypical pneumonia like SARS, are used to treat patients with new coronary pneumonia, which have obvious adverse side-effects and drug resistance. Antiviral drugs are known to cause high replication rates, which result in the viral resistant, weaken the host immune system, and only specifically inhibit a small proportion of the virus [3–5]. There is an urgent need to develop safe, effective and inexpensive potential targeted therapeutic drugs with low or minimal toxicity. The main protease (Mpro) of SARS-CoV-2 has been extensively investigated as a druggable target for COVID-19 due to its critical role in viral replication and transcription [6]. Mpro hydrolyzes polyproteins from multiple conserved sites, resulting in a variety of nonstructural proteins, such as RNA-dependent RNA polymerases and helicases, for assembly into new viruses. At the same time, Mpro also has a highly conserved substrate specificity, which is characterized by efficient cleavage in peptide fragments, including [(Leu, Phe, Met, Val)-Gln↓(Ser, Ala, Gly)] sequence (cleavage site indicated with ↓) [7]. The critical role of Mpro in the infection process and the highly conserved substrate specificity make it an important research target for SARS-CoV-2. There have been great efforts to find SARS-CoV-2 Mpro inhibitors as potential drugs for the treatment of SARS-CoV-2 [8, 9].

Chitosan is the unique known natural alkaline cationic polymer, which widely exists in marine crustaceans, and has the characteristics of excellent biocompatibility, antibacterial properties, biodegradability and broad-spectrum antiviral activity [10–12]. Chitosan has anti—Avian influenza A (H7N9) activity and its intranasal delivery can effectively activate mucosal immune responses, resulting in the increased levels of pro-inflammatory cytokines in bronchial and lung tissues [13]. Chitosan and its derivatives also have antiviral effects on the SARS-CoV-2, and can prevent and treat respiratory diseases caused by the virus without unnecessary toxic side effects. Chitosan occupies a unique position among natural polymers due to its antibacterial, antifungal and antiviral properties. It can inhibit the growth of various bacteria and fungi, and can also enhance the antiviral immune response [14]. It has been recently reported that β-chitosan prevents the attachment of angiotensin converting enzyme-2 (ACE2) to the receptor-binding domain (RBD) of the SARS-CoV-2 S protein, and β-chitosan can effectively attach to SARS-CoV-2 RBD or ACE2, blocking the binding of RBD to ACE2 for the reduction of SARS-CoV-2 infection [15–17]. These results provide us with new ideas and confidence to explore the effect and mechanism of CS with various molecular weights on the enzymatic activity of SARS-CoV-2 Mpro.

On the basis of the high homology between SARS-CoV-2 and SARS-CoV, we took SARS-CoV-2 Mpro as the research target and systematically investigated the effect of CS on the enzymatic activity of SARS-CoV-2 Mpro [18]. The interaction between CS and SARS-CoV-2 Mpro was studied by FRET, UV–Vis spectrometry, synchronous fluorescence spectroscopy, circular dichroism spectroscopy, and molecular docking technology. In addition, we explored the interaction mechanism of CS and Mpro. It will provide a theoretical basis for the application of CS to anti-SARS-CoV-2 and provide a new idea for the screening and application of anti-SARS-CoV-2 drugs.

## Materials and methods

### Materials

Ni^2+^ HisTrap prepacked column was obtained from GE healthcare. CS standards were purchased from Qingdao BZ Oligo Biotech company. SARS-CoV-2 Mpro activity fluorescence detection kit was obtained from Beyotime. Modified bradford method protein concentration determination kit was purchased from Shanghai Sangon.

### Protein expression and purification

Sequences encoding the domain of the SARS-CoV-2 Mpro (UniProtKB accession P0DTD1, residues 3264–3569) were optimized for the synthesis by WuHan Genecreat Biological Engineering [19]. We used the previously reported cloning strategy to produce authentic viral Mpro. A pET28a plasmid containing the SARS-CoV-2 his-tagged Mpro sequence was transformed into BL21 (DE3) Gold *E. coli* strain. Bacterial cultures were grown in 250 mL of LB/kana (Sangon, Shanghai, China, 100 μg/mL) media at 37^◦^C overnight with gentle shaking. Then, 1 L of LB/kana (100 μg/mL) was inoculated and incubated under the same condition until reaching OD = 0.6–0.8 at a wavelength of 600 nm and 0.5 mM of IPTG (Sangon, Shanghai, China) was added to the cell culture to induce the expression at 37^◦^C. After 4 h, the cells were collected by centrifugation for 10 min at 6,000 × g (Hitachi, Tokyo, Japan). The cell pellets were resuspended in PBS containing bacterial protein preparation lysate (Sangon Biotech, Shanghai, China). Cell rupture was achieved by sonication (Scientz, Ningbo, China) in ice. To remove cellular debris, the extract was centrifuged at 4^◦^C and 12,000 rpm for 30 min and filtered through a 0.22 μm-pore membrane. The supernatant was loaded onto Ni–NTA affinity column and eluted in an imidazole 20–500 mM gradient. The purified Mpro was stored in 50 mM Tris–HCl (pH 7.3, 2 mM β-ME).

### SARS-CoV-2 Mpro enzyme inhibition assay

FRET protease assay was performed to measure the inhibitory activity of compounds against the SARS-CoV-2 Mpro. The fluorogenic substrate (MCA-AVLQSGFR-Lys(Dnp)-Lys-NH_2_) was synthesized by Beyotime (Shanghai, China). The FRET-based protease assay was performed as follows [20]. The recombinant SARS-CoV-2 Mpro (1.5 µM at a final concentration) was mixed with serial dilutions of each CS in 80 µL assay buffer (50 mM Tris, pH 7.3, 1 mM EDTA) and incubated for 10 min. The reaction was initiated by adding 2 µL fluorogenic substrate with a final concentration of 20 µM. After that, the fluorescence signal at 320 nm (excitation) / 405 nm (emission) was immediately measured with a Thermo Varioskan LUX 3020–213 plate reader. Three independent experiments were performed. All experimental data were analyzed by using the GraphPad Prism software.

### UV–vis absorption

UV–vis spectra were determined according to the previous report [21]. Mpro (1.0 × 10^−5^ M) solution and CS solution (5.0 × 10^−3^ M) were used in the fluorescence experiment. The buffer used in the fluorescence experiment was 50 mM phosphate buffer (PB, pH 7.4). In the UV–vis spectra assay, Mpro was kept constant while varying the concentration of CS.

### Fluorescence experiment

The synchronous fluorescence spectra of Mpro (1.0 × 10^−5^ M) solution and CS solution were obtained with a FP-6500 (JASCO, Japan) fluorescence spectrophotometer as reported elsewhere [22]. The Δλ was set to 20 nm and the scan range was set to 220–690 nm. The buffer used in the fluorescence experiment was same with that of UV.

### Circular dichroism measurement

The far-UV CD measurements were performed with a J-810 spectrometer (JASCO, Japan) at room temperature under a nitrogen atmosphere with a band width of 1 nm. The concentrations of protein and CS were kept constant to ensure that the molar ratio of CS to protein was 1:10. The CD measurements of Mpro in the presence and absence of CS were made in the range of 200–250 nm. The CD result was expressed as a molar ellipticity, ([Θ]) in deg cm^2^ dmol^−1^, as defined below:

$$\left[\Theta\right]=\frac{\Theta_{\mathrm{obs}}}{10nlC_{\mathrm p}}$$

where obs is the CD in millidegree, *n* is the number of amino acid residues (306), *l* is the path length of the cell (0.1 cm), and *C*_*p*_ is the mole fraction. The helical content could be calculated from the [Θ] values at 208 nm using the following equation [23]:

$$\mathrm{\alpha }-\mathrm{helix\%}=\frac{{-\left[\Theta \right]}_{208}-4000}{33000-4000}\times 100$$

### Molecular docking

*Docking-based virtual screening:* The 2.16 Å crystal structure of SARS-CoV-2 Mpro was extracted from the Protein Data Bank (PDB ID: 6LU7) [24, 25]. The enzyme structure and ligand were subjected to an energy minimization step using the OPLS-2005 force-field. Molecular-docking was based on the docking protocols of Glide, followed by “Extra-Precision” mode (XP). The input compounds were assessed by using the docking-based virtual screening and filtered to final four optimized lead compounds based on the Glide-Gscores.

*Alanine Scanning:* Using the alanine scan in Schrödinger software, Glu166 was mutated to Ala166, the structure of the mutant protein was modeled, and the effect of Glu166 on the complex was explored with changes in the affinity.

## Results

### Inhibition of SARS-CoV-2 Mpro by CS

**Fig. 1:**
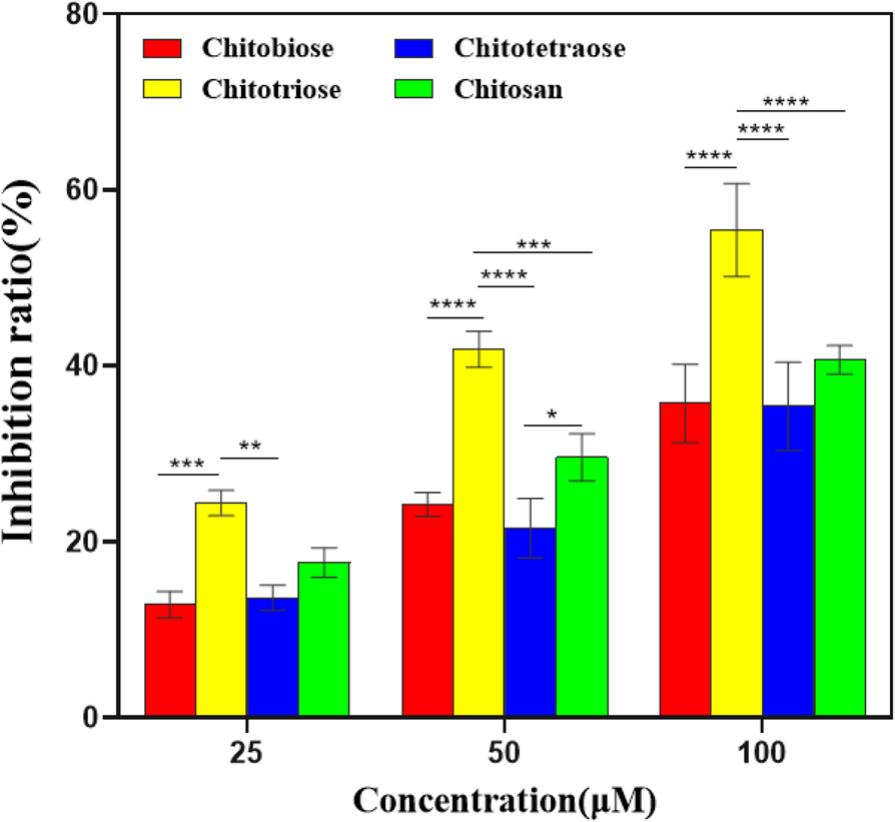
The inhibitory effect of chitooligosaccharides such as chitobiose, chitotriose, chitotetraose, and chitosan

FRET has been widely used to study the interaction between small molecules and enzymes [26, 27]. First, using the fluorescent chromophore and the highly conserved substrate specificity of protease, we investigated the effect of adding CS on Mpro enzyme activity. Figure 1 shows the effect of various concentrations of CS on the enzymatic activity of SARS-CoV-2 Mpro. The results showed that all of the four different molecular weights of CS at the concentrations of 25, 50 and 100 μM showed the inhibitory effect on SARS-CoV-2 Mpro, and the inhibition rate of chitotriose was significantly higher than that of other CS. Especially, when the concentration of chitotriose was 100 μM, the enzyme inhibition rate reached 55%. The inhibition of Mpro enzyme activity might be attributed to the combination of CS with the active site of Mpro, which changed the conformation of Mpro and interacted to form a complex. In order to explain the change of enzyme activity, a series of spectroscopic experiments and computer simulations were used to study the change of Mpro after the addition of CS.

### UV–Vis absorption spectroscopy

**Fig. 2:**
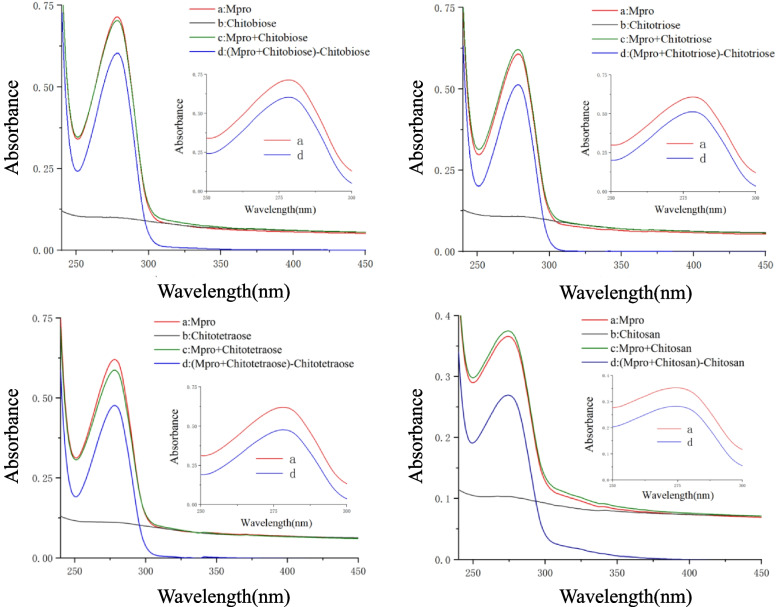
The absorption spectra of chitooligosaccharides bound to Mpro

The change in the UV–Vis absorption spectrum is caused by the formation of complexes [28]. UV–Vis absorption spectra can visualize the structural change of Mpro upon the complex formation with CS. Figure 2 shows the UV absorption spectra for the interaction between CS with various molecular weights and Mpro. Figure 2 shows that there are 4 curves: Mpro (curve a), CS with a different molecular weight (curve b), a mixture of Mpro and CS (curve c), the subtraction of CS from the mixed solution (curve d). Due to the transition of valence electrons, Mpro has a strong absorption peak at 280 nm, which is mainly generated by the π-π* transition of the benzene ring of the aromatic amino acid residue of Mpro. From the comparison of curves a and d, it can be found that the strong absorption peak of Mpro at 280 nm has been significantly changed by the addition of CS, indicating that the addition of CS changes the conformation of Mpro and the microenvironment around the aromatic amino acids. The scanning results of UV–Vis spectra confirmed the interaction between CS and Mpro. It shows that the addition of CS changes the conformation of Mpro, affects the microenvironment of aromatic amino acid residues, and then affects the enzymatic activity of Mpro.

### Fluorescence quenching

**Fig. 3:**
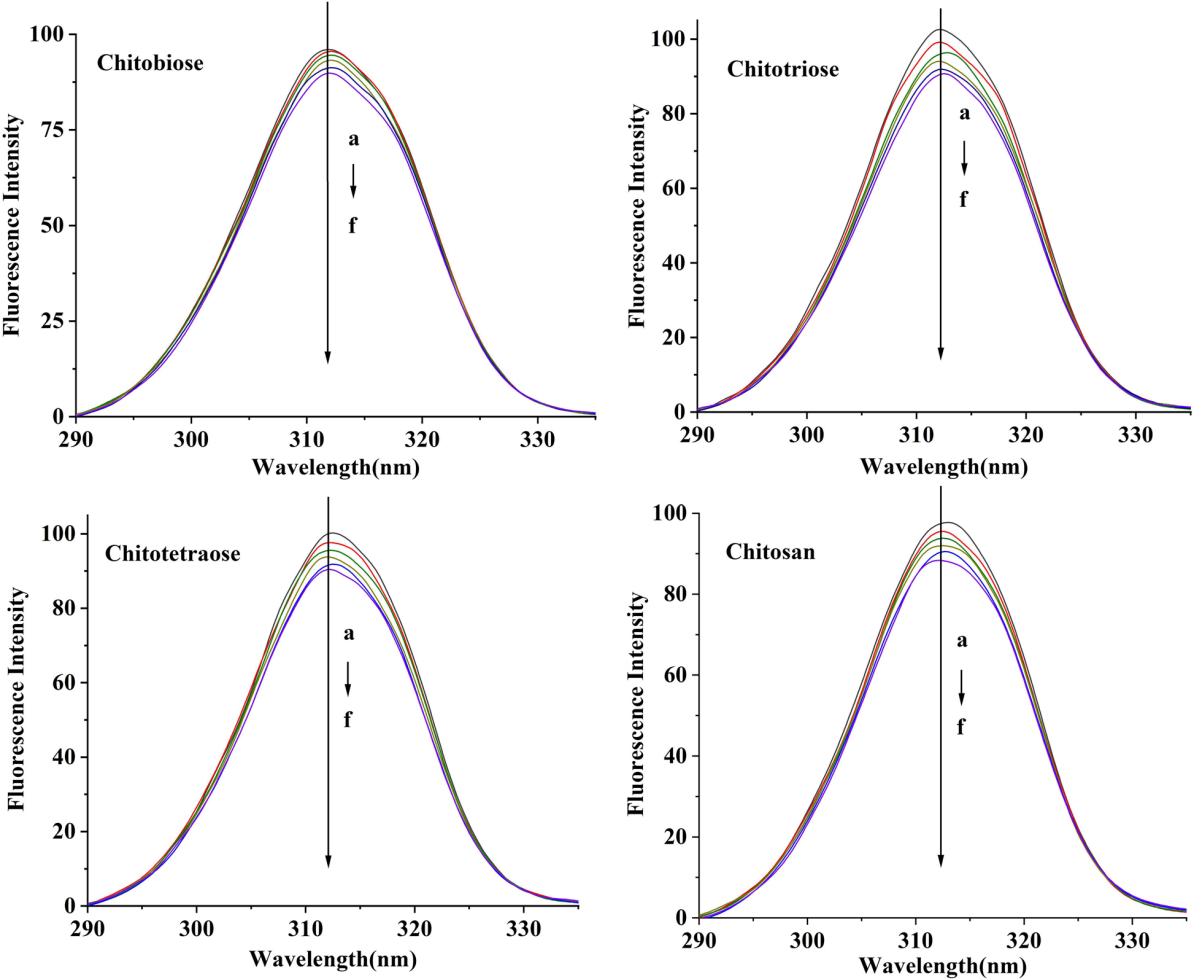
The fluorescence spectra of Mpro in the presence of chitooligosaccharides (pH = 7.4, λ_ex_ = 288 nm). In the figure, a to f represent chitooligosaccharides at concentrations of 0, 1.0, 2.0, 3.0, 4.0, and 5.0 × 10^−4^ mol/L, respectively

Synchronous fluorescence spectroscopy has been used to characterize the interaction of small molecules with proteins on the secondary structure, and to reflect the microenvironment information of protein fluorophores [22]. Under a specific Δλ, as other proteins, the aromatic amino acid residues of Mpro can emit endogenous fluorescence, which is often used as an effective indicator of protein structural changes. Figure 3 shows the effect of various molecular weights CS on the fluorescence emission spectrum of Mpro.

When the excitation wavelength is 288 nm, Mpro has a strong fluorescence at 330 nm. With increasing concentration of CS, the fluorescence intensity of Mpro gradually decreased, because the interaction of CS and Mpro made the conformational change of Mpro. The microenvironment of tyrosine residues resulted in the quenching of Mpro's endogenous fluorescence. Synchronous fluorescence spectra also proved the interaction between CS and Mpro, indicating that the addition of CS changed the conformation of Mpro, changed the microenvironment of tyrosine residues, and then possibly affected the Mpro enzyme activity. The results were well matched with that of UV–Vis.

### Circular dichroism

**Fig. 4:**
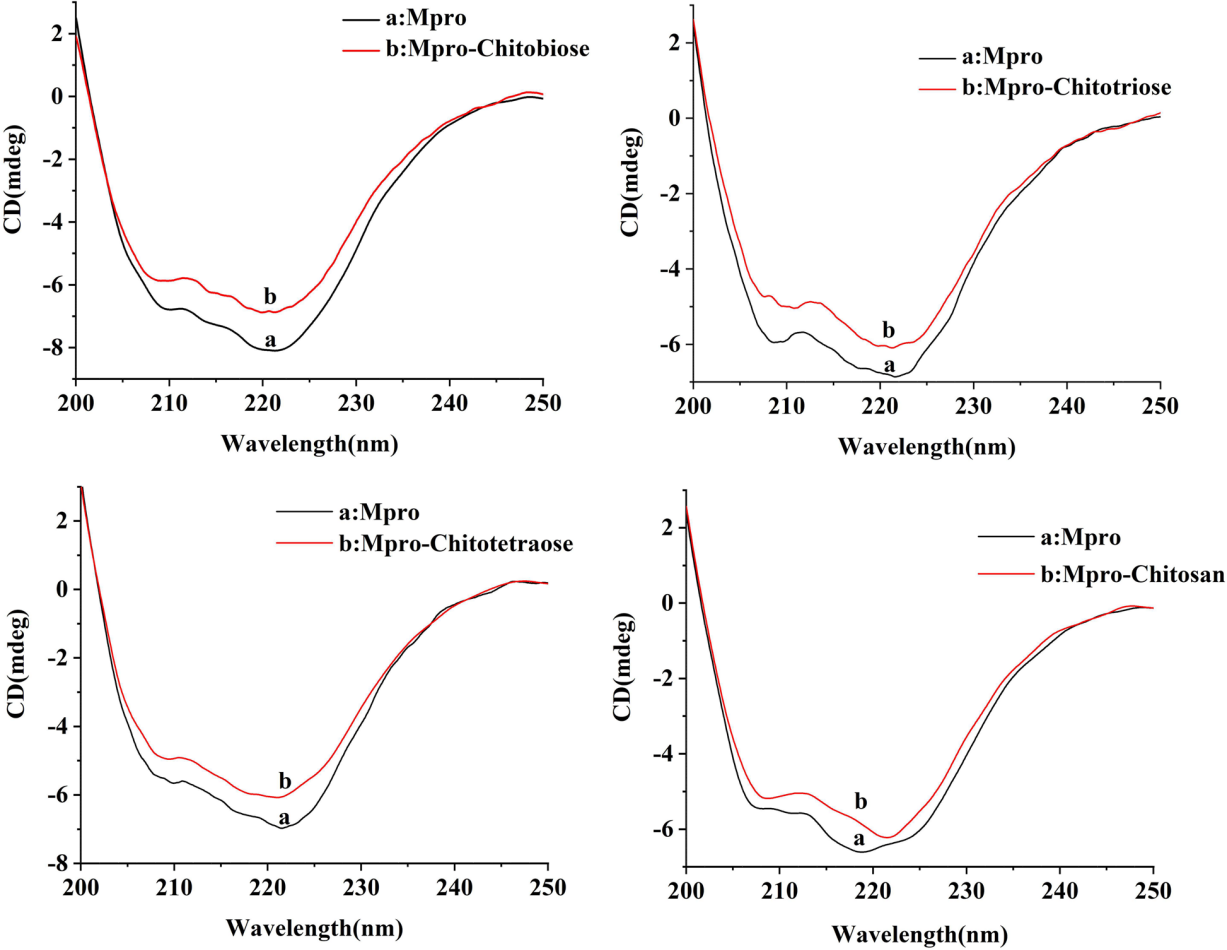
CD spectra in the absence and presence of chitooligosaccharides. The lower spectrum represents Mpro and the upper spectrum represents Mpro-chitooligosaccharides

CD is commonly used to characterize the structure of proteins, especially for the determination of conformational changes and alterations in secondary structure [29, 30]. The interaction of CS and Mpro can cause changes in the protein structure. The structural changes of CS on Mpro were explored by CD. Figure 4 shows the CD spectra of CS and Mpro. The CD spectrum of Mpro showed two distinct negative peaks at 208 and 220 nm, which was the typical of α-helix and consistent with the crystal structure. After the addition of CS, the amplitude of the negative peak was lowered, indicating that the helical structure of Mpro was affected, but the shape of the peak did not change and the α-helix was still the main secondary structure of Mpro.

The effect of CS on the conformation of Mpro can be determined according to the changes in the protein α-helix, β-sheet and random coil contents. In this study, to further explore the effect of four CS on the spatial structure of Mpro, we calculated the α-helix content of the four compounds. The calculated content of α-helix in Mpro with and without chitobiose was 45.7% and 40.4%, respectively, and the addition of chitobiose reduced the content of Mpro α-helix by about 5%. At the same time, as described in Table 1, it was found that the content of α-helix in Mpro was reduced to different degrees after the addition of four CS, respectively. Among them, chitotriose had the greatest effect on the structure of Mpro, and the content of α-helix decreased by 11.8%. CD showed that the four kinds of CS interacted with Mpro, and the partial arrangement of Mpro led to the loosening and reduction of the α-helix structure, which changed the spatial structure of Mpro, and then possibly inhibited the enzymatic activity of Mpro. In order to study the mechanism of action of the complex in more detail, we carried out the computational simulation for molecular docking in the following.

**Table 1:** The effect of chitooligosaccharides on Mpro α-helix

CS	Chitobiose	Chitotriose	Chitotetraose	Chitosan
Without	45.66%	39.86%	37.19%	37.42%
With	40.35%	28.06%	31.35%	31.17%

### Molecular docking

**Fig. 5:**
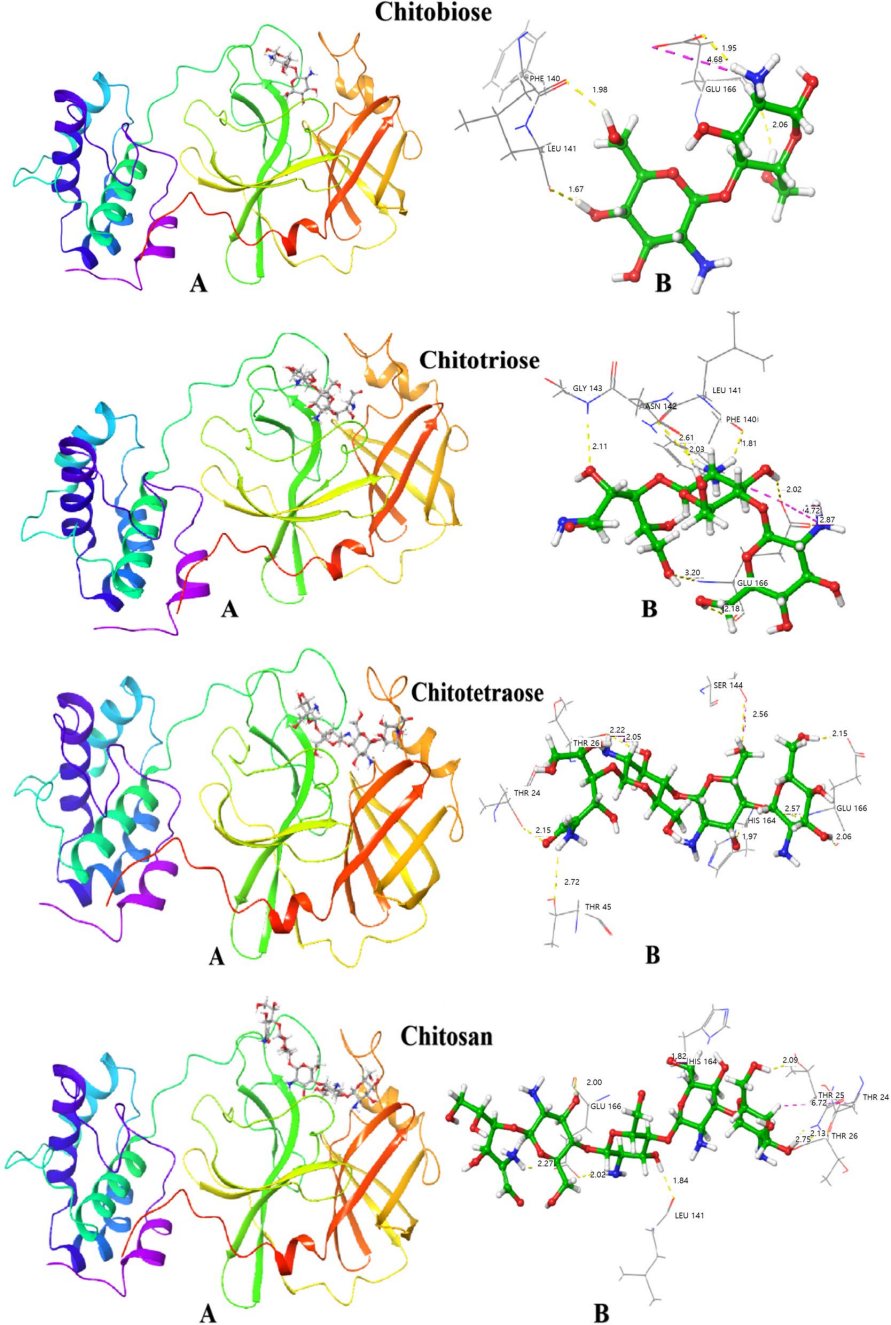
The molecular modeling for the interaction between Mpro and chitooligosaccharides (CS). **A** Mpro is represented in the solid ribbon. **B** CS is displayed in a stick model and amino acid residues are displayed in the line model. The dotted yellow line denotes hydrogen bonding and the dotted purple line denotes salt-bridge

The molecular interaction between Mpro and CS in the key amino acids was further explored by computational simulation. Molecular docking refers to the use of computers to simulate the mechanism of action of small molecule ligands and protein receptors in a fixed network lattice, providing a theoretical background for the subsequent experiments [31]. CS with various molecular weights (chitobiose, chitotriose, chitotetraose, and chitopentaose) were used as ligands and Mpro was used as the receptor for the molecular docking. Figure 5 shows the optimal docking conformation, which reveals the CS with different molecular weights have been inserted into the active site of Mpro and interacted with amino acid residues (such as Thr, Glu, His, Ser, etc.). The docking scores and specific amino acid residues are described in Table 2. The results showed that all of chitobiose, chitotriose, chitotetraose and chitopentaose had hydrogen bonding interactions with Mpro protein, and the hydrogen bonding was an important force to maintain the stability of CS and Mpro complex. At the same time, chitobiose and chitotriose can also interact with Mpro via salt bridge, which is also very critical for the stability of the complex. By comparison, it was found that chitosan had the highest docking score, probably because it contained more hydroxyl groups and could form a more stable complex with Mpro. It is worth of noting here that the Mpro amino acid Glu166 interacts with chitobiose, chitotriose, chitotetraose, and chitopentaose. Glu166 is also a key amino acid in the structure of Mpro, which plays a key role in maintaining the structural stability. It is preliminarily speculated that Glu166 plays an important role in stabilizing the binding of CS to Mpro. To explore the important role of Glu166 on the affinity of CS-Mpro complexes, we carried out alanine scanning in the following.

**Table 2:** The interaction between Mpro and chitooligosaccharides

	Glide-gscore (kcal/mol)	H-bond	Salt-Bridge
Chitobiose	-9.713	Phe140, Leu141, Glu166	Glu166
Chitotriose	-11.681	Phe140, Leu141, Asn142, Gly143, Glu166	Glu166
Chitotetraose	-9.836	Thr24, Thr26, Thr45, Ser144, His164, Glu166	/
Chitosan	-12.400	Thr24, Thr25, Thr26, Leu141, His164, Glu166, Pro168	/

### Alanine scanning

**Fig. 6:**
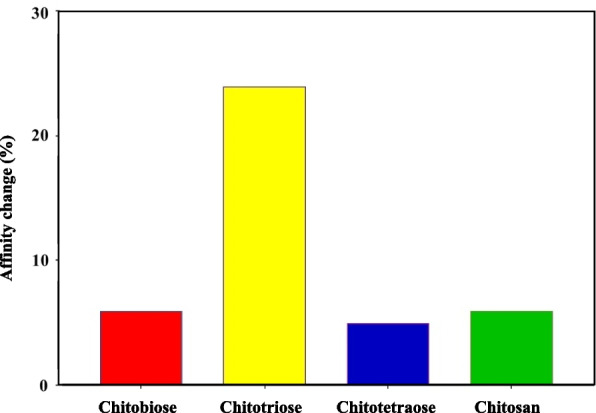
The affinity changes by E166A mutation in chitooligosaccharides

Key amino acids play an important role in the formation of protein–ligand complexes and affect the affinity of the complexes [32]. Molecular docking showed that Glu166 as a key amino acid was involved in the formation of hydrogen bonds and salt bridge interactions for the interaction between CS and Mpro. To verify the effect of Glu166 on the affinity of the complex, Glu166 was mutated to Ala166 by alanine scanning. Figure 6 shows the alanine scanning results. The affinity of mutated Mpro with the four CS was reduced, indicating that Glu166 was the key amino acid of Mpro and played a key role in the structure. Furthermore, the Glu166 mutation had the greatest effect on the affinity of the CS-Mpro complex. In addition to forming multiple hydrogen bonds, the negatively charged carboxyl group of Glu166 can form a salt bridge with the amino group of chitotriose. In a word, the mutation of Glu166 to Ala166 might be the reason for the sudden change of affinity.

The Mpro structure has a Cys-His catalytic dyad with a substrate-binding site located in the cleft between domain I (residues 8–101) and domain II (residues 102–184). The amino acid Glu166 of Mpro locates in this gap, and is in an antiparallel sheet in the structure, which is involved in the formation of key sites. The position of Glu166 is particularly sensitive and the affinity of the complex changes significantly after mutation. Glu166, as the key amino acid for Mpro binding, can be used as a new target for the development of target-specific Mpro inhibitors.

## Discussion

A variety of carbohydrate polymers including hyaluronate (HA) and chitosan have been widely investigated for drug delivery applications [33–35]. Especially, HA has been used for liver targeting, ocular and transdermal drug delivery applications [33]. In addition, chitosan has been used for oral drug delivery applications with strong interaction to the tight junction of intestines [35]. In this study, the interaction and action mechanism of CS to targeting SARS-CoV-2 Mpro were investigated by enzymatic activity analysis, spectroscopy and computational simulation. The recombinant protein SARS-CoV-2 Mpro was successfully purified via prokaryotic expression. Four kinds of CS reduced the enzymatic activity in a concentration-dependent manner, and chitotriose showed a significant inhibitory effect on the Mpro enzymatic activity. Various spectroscopic studies have shown that all four CS can interact with Mpro. CS can change the microenvironment of Mpro amino acid residues, thereby affecting the spatial structure of Mpro and the activity of Mpro enzyme. Chitosan had the greatest impact on the spatial structure of protein. Chitotriose might be the most potential antiviral compound. The complexes were mainly formed by hydrogen bonds and salt bridges to form stable complexes. At the same time, Glu166, as a key amino acid, played an important role in maintaining the Mpro-CS complex, which would be used as a target for Mpro inhibitors, providing a new direction for the development of targeted Mpro inhibitors. Further experiments will be carried out to investigate the effect of CS on the anti-SARS-CoV-2.

## Conclusion

On the basis of the high homology between SARS-CoV-2 and SARS-CoV, this study took SARS-CoV-2 Mpro as the research target to investigate the effect of CS with various molecular weights on the activity of SARS-CoV-2 Mpro and the interaction between them. The results showed that four kinds of CS with different molecular weights could reduce the activity of Mpro and form stable complexes with Mpro through hydrogen bond and salt bridge interaction. In this interaction, Glu166 appeared to be the key amino acid. This study provides a theoretical basis for the development of targeted Mpro inhibitors, and provides a new idea for the screening and application of anti-novel coronavirus drugs.

## Data Availability

Not applicable.

## Abbreviations

ACE2: Angiotensin converting enzyme-2
CD: Circular dichroism
COVID-19: Coronavirus disease 2019
CS: Chitooligosaccharide
FRET: Fluorescence resonance energy transfer
Mpro: Main protease
RBD: Receptor-binding domain
SARS-CoV-2: Severe acute respiratory syndrome coronavirus
